# 14-3-3η Proteins as a Diagnostic Marker, Disease Activation Indicator, and Lymphoma Predictor in Patients with Primary Sjögren Syndrome

**DOI:** 10.34172/aim.2023.85

**Published:** 2023-10-01

**Authors:** Ahmet Kor, Merve Yalçın, Şükran Erten, Yüksel Maraş, Esra Fırat Oğuz, İsmail Doğan, Ebru Atalar, Salih Başer, Özcan Erel

**Affiliations:** ^1^Department of Rheumatology, Aksaray Education and Research Hospital, Aksaray, Turkey; ^2^Department of Internal Medicine, Ankara Bilkent City Hospital, Ministry of Health, Ankara, Turkey; ^3^Department of Rheumatology, Faculty of Medicine Ankara Bilkent City Hospital, Ankara Yıldırım Beyazıt University, Ankara, Turkey; ^4^Department of Rheumatology, Ankara Bilkent City Hospital, Health Sciences University, Ankara, Turkey; ^5^Department of Medical Biochemistry, Ankara Bilkent City Hospital, Ministry of Health, Ankara, Turkey; ^6^Department of Rheumatology, Ankara Bilkent City Hospital, Ministry of Health, Ankara, Turkey; ^7^Department of Internal Medicine, Faculty of Medicine Ankara Bilkent City Hospital, Ankara Yıldırım Beyazıt University, Ankara, Turkey; ^8^Department of Medical Biochemistry, Faculty of Medicine Ankara Bilkent City Hospital, Ankara Yıldırım Beyazıt University, Ankara, Turkey

**Keywords:** 14-3-3η protein, Lymphoma, Primary Sjögren syndrome

## Abstract

**Background:**

Primary Sjögren syndrome (PSS) is a chronic, autoimmune, and lymphoproliferative disease of the connective tissue. In patients with PSS, the risk of developing B-cell non-Hodgkin lymphoma (NHL) increases dramatically, with a prevalence of approximately 5%. The 14-3-3 protein isoforms are phospho-serin/phospho-threonine binding proteins associated with many malignant diseases. This study aimed to evaluate the relationship between disease activity parameters and markers predicting lymphoma development in patients with PSS and 14-3-3η proteins.

**Methods:**

This study was designed as an analytical case-control study. A total of 57 PSS patients and 54 healthy volunteers were included in the study. The European League Against Rheumatism (EULAR) Sjögren syndrome disease activity index (ESSDAI) was used to assess systemic disease activity in PSS. Receiver operating characteristic (ROC) analysis was used to test the diagnostic accuracy measures of the analytical results. Multivariable linear regression analysis was used to evaluate the effects of independent variables on the 14-3-3η protein.

**Results:**

The 14-3-3η protein serum levels were found to be significantly higher in PSS (2.72 [2.04-4.07]) than healthy controls (1.73 [1.41-2.43]) (*P*<0.0001). A significant relationship was found between 14-3-3η protein levels and ESSDAI group (β=0.385, 95%CI=0.318-1.651, *P*=0.005), hypocomplementemia (C3 or C4) (β=0.223, 95% CI=0.09-1.983, *P*=0.048) and purpura (β=0.252, 95% CI=0.335-4.903, *P*=0.022), which are accepted as lymphoma predictors. A significant correlation was found between PSS disease activity score ESSDAI and 14-33η protein (β=0.496, 95% CI=0.079-0.244, *P*=0.0002).

**Conclusion:**

14-3-3η proteins are potential candidates for diagnostic marker, marker of disease activity, and predictor of lymphoma in PSS patients.

## Introduction

 Primary Sjögren syndrome (PSS) is a chronic, autoimmune, and lymphoproliferative disease of the connective tissue characterized by development of lacrimal and salivary glands dysfunction due to lymphocytic infiltration of the exocrine glands.^1^ High serum levels of anti-SSA/Ro autoantibody, whose main target is autoantigen Ro52, are characteristic of PSS.^2^ However, similar to other autoantibodies shown to be associated with PSS, the role of these autoantibodies in the pathogenesis of the disease is unclear. Ro52 inhibits the synthesis of inflammatory cytokines, so anti-Ro52 autoantibodies inhibit the effects of Ro52 in patients with PSS. This results in increased production of cytokines that contribute to the pathogenesis of PSS.^3^ Formation of mucosal-associated lymphoid tissue (MALT) in exocrine glandular tissues is the main pathophysiological feature of PSS.^4,5^ Because of this feature, PSS has been also called “autoimmune exocrinopathy” or “autoimmune epitheliitis”.^6^ The risk of developing B-cell non-Hodgkin lymphoma (NHL), which represents the leading cause of increased mortality in patients with PSS, is significantly increased, and its prevalence is approximately 5%.^7^

 Markers (laboratory, pathological and clinical) that may have predictive value in developing lymphoma in PSS have been investigated since the 1970s. Clinically, the best predictors for lymphoma development are mixed cryoglobulinemia and/or cryoglobulinemic vasculitis, persistent salivary gland swelling,^4,8,9-17^ and the presence of skin purpura and low C4 level, which may be associated with cryoglobulinemia.^18^ Cryoglobulinemia and/or cryoglobulinemic vasculitis and persistent salivary gland swelling are closely related to other pathological, laboratory, and clinical predictors of lymphoma in PSS.^11,15,19^ Other additional pathological, laboratory, and clinical predictors of lymphoma in PSS include organ involvement associated with cryoglobulinemic vasculitis (peripheral neuropathy, glomerulonephritis),^20^ high MALT involvement in salivary gland histopathology, presence of monoclonal gammopathy and RF positivity^4-6,8,21^ and specific idiotypes that play a role in the pathophysiology of cryoglobulinemia.^18^ In addition, lymphopenia, neutropenia, elevated free immunoglobulin light chains, increased serum beta-2 microglobulin, lymphadenopathy, splenomegaly, genetic abnormalities, cytokines, chemokines, growth factors, monoclonal B lymphocyte expansion in metachronous tissue histopathology and more recently, the European League Against Rheumatism (EULAR) Sjögren syndrome disease activity index (ESSDAI) are recommended as predictive markers of lymphoma in PSS.^4,7,11,17,19-22^

 The 14-3-3 proteins consist of phospho-serine/phospho-threonine-binding isoforms that are associated with different protein groups such as phosphatases, kinases, transcription factors, and transmembrane receptors.^23^ The 14-3-3 proteins can be found in all systems of eukaryotic organisms and interact with numerous functional molecules, usually phosphorylated, to regulate numerous physiological processes such as cell proliferation, intracellular protein trafficking, apoptosis, signal transduction, growth, stress responses, and regulation of metabolism. The 14-3-3 proteins, which have seven isoforms (β, γ, ε, η, σ, θ, and ζ), have a structure consisting of a highly conserved protein family.^23^ Evidence shows that dysregulations in the 14-3-3 proteins are associated with essential diseases such as malignant diseases, neurodegenerative diseases, giardia intestinalis infection, and rheumatoid arthritis (RA).^24-28^ Abnormal expression of the 14-3-3 proteins strongly correlates with many malignant diseases, and the 14-3-3 proteins can target oncogenic proteins.^29-31^ The association of the 14-3-3 proteins with malignant diseases varies according to the isoform and the tissue in which it is expressed. In addition to the association of the 14-3-3ζ protein with lung, breast, prostate, ovarian and gastric cancers,^32^ it is also associated with chemotherapy resistance and poor prognosis in diffuse large B-cell lymphoma and extranodal NK/T-cell lymphoma.^33-35^ This association with malignancies is caused by increased cancer cell survival and Akt activation due to the interaction between the p85 regulatory subunit of PI3 kinase and 14-3-3ζ.^36^ Among other isoforms, 14-3-3β is associated with gastric cancer,^37^ while 14-3-3σ is associated with breast cancer and chronic myeloid leukemia.^38,39^ Studies have shown that the 14-3-3σ isoform, which can exhibit both pro-oncogenic and tumor-suppressive properties, is associated with c-Abl and proteins related to malignancy development, such as Raf1, p53, Cdc25, Bad, HDAC, and FOXO.^40,41^ The 14-3-3η protein isoform, whose relationship with malignancy is unknown, can bind parkin with nanomolar affinity and contributes to the development of autosomal recessive juvenile parkinsonism by inhibiting the ubiquitin-ligase activity of parkin after this binding.^23,42^ The 14-3-3η proteins are also associated with RA, joint erosion in RA, and secondary Sjogren’s syndrome (SSS) due to systemic lupus erythematosus (SLE).^25,43-46^

 It is essential to predict the development of lymphoma, the most severe complication resulting from PSS. Great efforts are being made to search for new markers that may predict lymphoma in PSS. Although many features indicate the development of lymphoma in PSS, the current titles in this area still need to be improved, and the need for new predictors persists. The relationship of the 14-3-3η protein with PSS is unknown. In this study, we aimed to investigate the usability of the 14-3-3η proteins as diagnostic test, disease activation indicator, and lymphoma predictor in PSS.

## Materials and Methods

###  Study Design

 The design of this study was prepared as an analytical case-control study. A total of 57 PSS patients, 48 females and nine males, were diagnosed according to the 2016 American College of Rheumatology/EULAR classification criteria,^47^ followed in the rheumatology department of Ankara Bilkent City Hospital, and were included in the study. The sample size required for optimal comparison of serum 14-3-3ƞ protein levels between PSS and health controls was determined by power analysis. A total of 54 healthy volunteers, 43 females and 11 males, were included in the control group. Pregnancy, active infection, active or former malignancy, and other rheumatological diseases except for PSS were accepted as exclusion criteria. Superficial and abdominal ultrasonography (USG) for detection of lymphadenopathy and splenomegaly; pulmonary function test, carbon monoxide diffusion test, or high-resolution computerized tomography for detection of interstitial lung disease; urine microscopy and renal biopsy histopathology for the detection of glomerulonephritis; neurological examination and electromyography to detect peripheral neurologic involvement; joint examination and joint USG to detect arthritis; physical examination and USG of the parotid gland to detect parotid gland swelling, and skin examination for the detection of purpura were used to screen for systemic organ involvement in PSS patients. ESSDAI^48^ was used to assess systemic disease activity in patients with PSS. According to this index, a total of 12 areas, including 11 sites related to organ involvement and one biological place reflecting B cell activity, were examined in patients. Patients were divided into ESSDAI groups that define systemic disease activity as low (5 > ), moderate ( > 5 and < 14), and severe ( > 14) according to the scores given. Individuals with diabetes mellitus, hypertension, chronic lung disease, or chronic heart disease were considered positive for the presence of comorbid diseases. All patients included in the study gave informed consent. Dates are indicated as DD/MM/YYYY.

###  Obtaining Sample Samples and Calculating 14-3-3 ƞ Values

 After venous blood samples were taken into vacuum tubes and centrifuged at 1300 × g for 10 minutes, the obtained sera were divided into Eppendorf tubes and stored at -80 °C until the analysis time. Human 14-3-3ƞ protein levels were measured with an ELISA kit (Fine Test, Wuhan, China; catalog no: EH2534; lot no: H2534G109 E) using the quantitative sandwich enzyme immunoassay technique. Optical density (OD) calculation was done by a spectrophotometric method using a microplate reader at 450 nm. OD value and human 14-3-3ƞ protein level concentrations were measured proportionally. Human 14-3-3ƞ protein concentrations were calculated by comparing the OD of the samples with the standard curve. The detection range of the test was 1.625–40 ng/mL, intra-assay precision < 8%, and interassay accuracy < 10%.

###  Statistical Analysis

 The Kolmogorov-Smirnov test and Q-Q plot, box plot, and histogram graphs were used to determine the normal distribution in continuous variables. Descriptive statistics were presented as mean and standard deviation (mean ± SD) for normally distributed variables and median (interquartile range [IQR], [25%-75%]) for non-normally distributed variables. The Mann-Whitney U test was used for non-normally distributed variables, and ındependent samples *t *test was used for normally distributed variables to determine the statistically significant differences in pairwise comparisons between groups. Spearman correlation analysis was used to determine the correlation between study parameters. Comparisons between multiple groups after the Bonferroni correction were made with the One-way ANOVA post-hoc Tukey test for normally distributed quantitative variables and the independent samples Kruskal-Wallis test for non-normally distributed quantitative variables. Multivariable linear regression analysis was used to evaluate the effects of independent variables on the 14-3-3η protein. The Chi-square and Fisher’s exact tests were used to compare categorical data. Receiver operating characteristic (ROC) analysis was used to test the diagnostic accuracy measures of the indexes, and results are shown with area under curve (AUC) and 95% confidence intervals (CIs). Youden’s index was used to determine the optimum cut-off value, and diagnostic accuracy criteria were presented. The accepted significance level for the *P *value was < 0.05 cut-off point in pairwise comparisons, while in multiple comparisons, the evaluation was made after Bonferroni correction. Statistical analyses were made using the Statistical Packages for the Social Sciences (SPSS) version 22.0.

## Results

###  Patients and Control Group

 Totally, 57 PSS patients with a mean age of 53.07 ± 9.43 years and 54 healthy volunteers with a mean age of 50.05 ± 14.46 years were included in the study. Age, gender, body mass index, presence of comorbid disease, and smoking rate were found to be similar between the PSS and control groups (*P* > 0.05). The median values of C-reactive protein (CRP) and erythrocyte sedimentation rate (ESR) were found to be similar between the groups. In contrast, the median values of the 14-3-3ƞ protein were significantly higher in the PSS group (2.72 [2.04-4.07]) than the controls (1.73 [1.41-2.43]) (*P* < 0.0001). The comparison between the groups in terms of age, gender, smoking, body mass index (BMI), presence of comorbid disease, and laboratory parameters is shown in Table 1.

**Table 1:** Comparison of Demographic Characteristics and Laboratory Parameters between PSS and Control Groups

**Parameters**	**PSS Group**	**Control Group**	***P*** ** Value**
Gender female/male, n	48\9, 57	43\11, 54	0.336
Age, mean ± SD (years)	53.07 ± 9.43	50.05 ± 14.46	0.192
Body mass index, mean ± SD	24.4 ± 4.02	25.9 ± 5.7	0.295
Presence of comorbid disease			
Diabetes mellitus	4	6	0.341
Hypertension	7	3	0.123
Chronic obstructive pulmonary disease	3	1	0.418
Coronary artery disease	2	4	0.386
Smoking, n (%)	5 (8.7)	3 (5.5)	0.328
Hemoglobin, mean ± SD [ × 10^9^/L]	13.41 ± 1.10	14.35 ± 1.37	0.85
Platelets, mean ± SD [ × 10^9^/L]	280.60 ± 84.07	272.71 ± 64.20	0.17
WBC, mean ± SD [ × 10^9^/L]	6.47 ± 2.05	6.56 ± 1.88	0.816
Neutrophil, mean ± SD [ × 10^9^/L]	4.10 ± 1.65	5.26 ± 1.02	0.404
Lymphocyte, mean ± SD [ × 10^9^/L]	1.35 ± 0.51	1.78 ± 0.63	0.086
Creatinine, mean ± SD [mg/dL]	0.66 ± 0.14	0.70 ± 0.11	0.14
ALT, mean ± SD [U/L]	18.87 ± 9.1	20.86 ± 9.78	0.21
LDH, mean ± SD [U/L]	211.91 ± 47.84	190.92 ± 33.3	0.09
CRP, median (IQR) [mg/L]	3.2 (1.8-9.5)	1.5 (1.6-7.7)	0.157
ESR, median (IQR) [mm/h]	13(7-19)	9.5(6-14)	0.074
Spot urine protein/creatinine ratio [mg/g]	180 ± 42.2	173 ± 36.8	0.231
14-3-3ƞ protein, median (IQR) [ng/mL]	2.72 (2.04-4.07)	1.73 (1.41-2.43)	< 0.0001

PSS, Primary Sjogren’s syndrome; WBC, White blood cells; ALT, Alanine aminotransferase; LDH, Lactate dehydrogenase; CRP, C-reactive protein; ESR, Erythrocyte sedimentation rate.

###  PSS Patients and Disease Characteristics

 The median duration of disease in the PSS group was 7 (5-11) years. AntiSSA/R052 antibody positivity was found in 43 patients (75.4%), while antiSSB antibody positivity was found in 26 patients (45.6%). The disease activity score was determined as an ESSDAI median value of 3 (2.5-14.5). Among the parameters predicting lymphoma in PSS, antiSSA/R052 (75.4%), antiSSB (45.6%), RF (19%), and hypocomplementemia (26%) were the most common findings, while peripheral neurological involvement (3.5%) and parotid gland enlargement (3.5%) were the least detected findings. The demographic, clinical, and laboratory data of the patients in the PSS group are shown in Table 2.

**Table 2:** Demographic, Clinical, and Laboratory Data of the Patients in the PSS Group

**Parameter**	**Value**
Disease duration, median (IQR) [years]	7 (5-11)
Lymphadenopathy, n (%)	7 (12.2)
Splenomegaly, n (%)	3 (5.2)
İnterstitial lung disease, n (%)	10 (17.5)
Glomerulonephritis, n (%)	3 (5.2)
Peripheral neurological involvement, n (%)	2 (3.5)
Central neurological involvement, n (%)	0 (0)
Arthritis, n (%)	4 (7)
Hypocomplementemia, (C3 or C4), n (%)	15 (26)
Hypocomplementemia, (C3), n (%)	13 (22)
Hypocomplementemia, (C4), n (%)	6 (10.5)
Monoclonal gammopathy, n (%)	3 (5.2)
Purpura, n (%)	3 (5.2)
Parotid gland enlargement, n (%)	2 (3.5)
Leukopenia, n (%)	3 (5.2)
Neutropenia, n (%)	2 (3.5)
Lymphopenia, n (%)	11 (19.2)
Anti-SSA/R052 positivity, n (%)	43 (75.4)
Anti-SSB positivity, n (%)	26 (45.6)
RF positivity, n (%)	19 (33)
ESSDAI, median (IQR)	3 (2.5-14.5)
RF, median (IQR) [IU/mL]	10 (5.5-21)
C3, median (IQR) [g/L]	1.19 (1.08-1.3)
C4, median (IQR) [g/L]	0.22 (0.19-0.27)
IgG, median (IQR) [g/L]	12.4 (10.5-14.5)
IgM, median (IQR) [g/L]	0.49 (0.1-1.02)

PSS, Primary Sjogren’s syndrome; RF, Rheumatoid factor; C3, Complement-3; C4, Complement-4; IgG, Immunoglobulin G; IgM, Immunoglobulin M; ESSDAI, EULAR Sjogren’s Syndrome Disease Activity İndex.

 No significant difference was found between those with (2.58 [2-3.6]) and without (3.85 [2.57-4.73]) ILD in the PSS groups in terms of 14-3-3ƞ protein levels (*P* = 0.142).

###  Relationship between 14-3-3 ƞ Protein and Drugs Used in PSS Medical Treatment

 There was no significant difference in serum 14-3-3ƞ protein levels between those who received any treatment as PSS treatment and those who did not receive the same treatment (*P* > 0.05). The comparison of 14-3-3ƞ protein levels according to medical treatment types in the PSS group is shown in Table 3.

**Table 3:** 14-3-3ƞ Protein Levels According to the Types of Medical Treatment used in the PSS Group

**Medical Therapy**		**n**	**14-3-3ƞ Protein Median (IQR) [ng/mL]**	***P*** ** Value**
Hydroxychloroquine	Yes	33	2.899 (2.03-4.15)	0.942
	No	24	2.62 (2.04-3.97)	
Corticosteroids	Yes	11	3.273 (2.56-4.37)	0.241
	No	46	2.690 (1.87-3.89)	
Methotrexate	Yes	3	2.051 (1.72-3.52)	0.353
	No	54	2.811 (2.16-4.14)	
Leflunomide	Yes	1	1.51	
	No	56	2.811 (2.08-4.10)	
Mycophenolate mofetil	Yes	4	3.637 (1.87-4.01)	0.660
	No	53	2.631 (2.04-4.14)	
Azathioprine	Yes	3	2.450 (1.64-3.58)	0.343
	No	54	2.098 (1.67-3.69)	
Rituximab	Yes	3	2.941 (2.16-3.84)	0.401
	No	54	3.639 (2.38-4.59)	
Pilocarpine	Yes	4	2.797 (2.16-4.35)	0.882
	No	53	2.84 (2.24-4.47)	

PSS, Primary Sjogren’s syndrome.

###  Sensitivity and Specificity for 14-3-3 ƞ Protein in PSS

**Figure 1:**
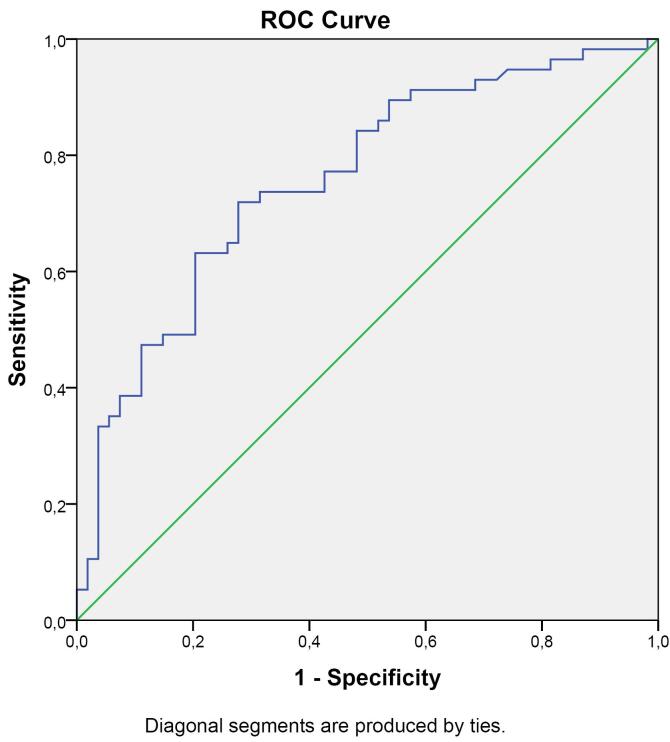
ROC Curve of 14-3-3ƞ Protein Levels in the PSS Group (AUC = 0.758, 95%CI = 0.669-0.848)

 In the ROC curve analysis comparing PSS patients and healthy controls in terms of 14-3-3ƞ protein levels, 95% CI = 0.669-0.848 and AUC = 0.758 were obtained. At a cut-off point of 1.741 ng/mL for 14-3-3ƞ protein, the ROC curve showed a sensitivity of 51.9% and a specificity of 84.2% (Figure 1).

###  Relationship between Lymphoma Predictive Markers and 14-3-3 ƞ Protein

 The median values of the 14-3-3ƞ protein were most closely related to the presence of hypocomplementemia (yes = 4.344 [3.52-5.98], no = 2.567 [1.81-3.3], *P* < 0.0001), ESDAI score (low = 1.838 [1.51-2.31], moderate = 3.165 (2.57-3.77), high = 4.222 (3.63-6.48), *P* < 0.0001) and RF positivity (yes = 3.855 [3.37-6.31], no = 2.567 [1.86-3.21], *P* = 0.001), which are predictors of lymphoma in PSS. A significant correlation was also found between the 14-3-3ƞ protein and hypergammaglobulinemia (yes = 3.855 [3.35-4.36], no = 2.578 [1.87-3.49], *P* = 0.009), AntiSSA/R052 positivity (yes = 3.273 [2.02--4.34], no = 2.348 [2.02-2.78], *P* = 0.04), AntiSSB positivity (yes = 3.476 [2.04-5.49], no = 2.573 [1.88-3.64], *P* = 0.016), and presence of purpura (yes = 4.607 [4.01-9.89], no = 2.631 [2-3.79], *P* = 0.024), lymphadenopathy (yes = 4.016 [3.18-4.37], no = 2.583 [1.87-3.89], *P* = 0.019) splenomegaly (yes = 4.299 [2.58-6.51], no = 2.658 [1.96-3.81], *P* = 0.024).

 In the comparison of 14-3-3ƞ protein levels in ESSDAI subgroups, a significant difference was found between low (1.838 [1.51-2.31]) and moderate groups (3.165 [2.57-3.77]) (*P* = 0.004) and between low (1.838 [1.51-2.31]) and high groups (4.222 [3.63-6.48]) (*P* < 0.0001) after Bonferroni correction. 14-3-3ƞ protein levels were found to be similar between moderate and high ESSDAI subgroups (respectively 3.165 [2.57-3.77], 4.222 [3.63-6.48], *P* = 0.045). Table 4 shows the relationship between lymphoma predictive markers and the 14-3-3ƞ protein in the PSS group.

**Table 4:** Relationship between Lymphoma Predictive Markers and the 14-3-3ƞ Protein in the PSS Group

**Parameters**		**n**	**14-3-3 ƞ Parametean (IQR) [ng/mL]**	***P*** ** Value**
Hypocomplementemia (C3 or C4)	Yes	15	4.344 (3.52-5.98)	< 0.0001
	No	42	2.567 (1.81-3.32)	
Hypocomplementemia (C4)	Yes	13	3.855 (3.21-5.03)	0.004
	No	44	2.573 (1.87-3.28)	
Hypocomplementemia (C3)	Yes	6	4.673 (4.04-5.58)	0.002
	No	51	2.583 (1.88-3.65)	
Hypergammaglobulinemia (IgG)	Yes	13	3.855 (3.35-4.36)	0.009
	No	44	2.578 (1.87-3.49)	
RF positivity	Yes	19	3.855 (3.37-6.31)	0.001
	No	38	2.567 (1.86-3.21)	
AntiSSA/R052 positivity	Yes	43	3.273 (2.02--4.34)	0.040
	No	14	2.348 (2.02-2.78)	
AntiSSB positivity	Yes	26	3.476 (2.04-5.49)	0.016
	No	31	2.573 (1.88-3.64)	
Purpura	Yes	3	4.607 (4.01-9.89)	0.024
	No	55	2.631 (2-3.79)	
Lymphadenopathy	Yes	7	4.016 (3.18-4.37)	0.019
	No	50	2.583 (1.87-3.89)	
Splenomegaly	Yes	3	4.299 (2.58-6.51)	0.024
	No	56	2.658 (1.96-3.81)	
ESSDAI groups	Low^*^	19	1.838 (1.51-2.31)	< 0.0001
	Moderate	23	3.165 (2.57-3.77)	
	High	15	4.222 (3.63-6.48)	
Parotid gland enlargement	Yes	2	2.657 (1.53-2.70)	0.664
	No	55	2.722 (2.05-4.13)	
Monoclonal gammopathy	Yes	3	3.273 (1.87-4.73)	0.748
	No	54	2.690 (2.04-4.04)	
Glomerulonephritis	Yes	3	3.855 (3.64-6.31)	0.093
	No	54	2.631 (2-4.04)	
Leukopenia	Yes	3	4.318 (4.22-4.37)	0.054
	No	54	2.631 (2-3.79)	
Neutropenia	Yes	2	4.299 (3.16-3.53)	0.118
	No	55	2.658 (2.04-3.85)	
Lymphopenia	Yes	11	3.831 (2.21-4.46)	0.157
	No	46	2.658 (1.88-3.65)	
Gender	Female	48	2.298 (1.65-3.43)	0.185
	Male	9	2.121 (1.62-2.56)	

PSS, Primary Sjogren’s syndrome; RF, Rheumatoid factor; C3, Complement-3; C4, Complement-4; IgG, Immunoglobulin G; ESSDAI, EULAR Sjogren’s Syndrome Disease Activity İndex. ^*^The group that creates statistical significance compared to the middle and high groups.

 Among the categorical lymphoma predictor subgroups with a significant difference in terms of the 14-3-3ƞ protein in Table 4, those affecting the 14-3-3ƞ protein independent variable were investigated by multivariable linear regression analysis. According to the model shown in Table 5, the ESSDAI group (β = 0.385, 95% CI = 0.318-1.651, *P* = 0.005), hypocomplementemia (C3 or C4) (β = 0.223, 95% CI = 0.09-1.983, *P* = 0.048) and purpura (β = 0.252, 95% CI = 0.335-4.903, *P* = 0.022), which are independent categorical variables, were found to positively and significantly affect the dependent variable, the 14-3-3ƞ protein.

**Table 5:** Multivariable Linear Regression Results between the 14-3-3ƞ Protein and some Predictors of Lymphoma

**Parameters**	**Unstandardized Coefficients** **(β-value)**	**Std. Error**	**Standardized Coefficients** **(β-value)**	**95 % CI ** **(Lower-Upper Bound)**		**VIF**	***P*** ** Value**
ESSDAI groups	0.984	0.331	0.385	0.318	1.651	1.873	0.005
AntiSSA/R052 positivity	-0.133	0.516	-0.029	-1.172	0.905	1.424	0.797
AntiSSB positivity	0.591	0.475	0.150	-0.365	1.348	1.618	0.220
Hypergammaglobulinemia (IgG)	-0.317	0.543	-0.068	-2.633	0.597	1.496	0.561
Hypocomplementemia (C3 or C4)	0.996	0.491	0.223	0.09	1.983	1.347	0.048
RF positivity	0.805	0.483	0.193	-0.167	1.777	1.497	0.102
Splenomegaly	1.700	1.165	0.193	-0.651	4.044	1.953	0.151
Lymphadenopathy	-0.742	0.857	-0.143	-2.467	0.982	2.285	0.391
Purpura	2.214	0.934	0.252	0.335	4.903	1.255	0.022
**14-3-3 ƞ protein**: Multiple R = 0.760; R^2^ = 0.578; Adjusted R^2^ = 0.497; Standart error = 1.405, F (9,47) = 7.143, *P* < 0.0001.							

CI, Confidence interval; VIF, Variance ınflation factor; RF, Rheumatoid factor; C3, Complement-3; C4, Complement-4; IgG, Immunoglobulin G; ESSDAI, EULAR Sjogren’s Syndrome Disease Activity Index.

###  Correlation between the 14-3-3 ƞ Protein and other Study Parameters

 While the highest correlation was found between 14-3-3ƞ protein levels and ESSDAI score (r = 0.537, *P* < 0.0001), a significant correlation was found between the 14-3-3ƞ protein and RF (r = 0.394,* P* = 0.002), AntiSSB (r = 0.355, *P* = 0.007), AntiSSA/RO52 (r = 0.311, *P* = 0.018) and IgG (r = 0.427, *P* = 0.001). Table 6 shows the correlation analysis results between the 14-3-3ƞ protein and other study parameters.

**Table 6:** Correlation Analysis Results between the 14-3-3ƞ Protein and some Study Parameters

**Parameter ** **r (p)**	**Age**	**Disease Duration**	**CRP Level**	**C3 Level**	**C4 Level**	**IgG Level**	**AntiSSA\** **RO52 Level**	**AntiSSB Level**	**RF Level**	**ESSDAI Score**
14-3-3 ƞ protein	-0.053 (0.697)	-0.017 (0.901)	-0.071 (0.594)	-0.242 (0.070)	-0.104 (0.439)	0.427 (0.001)	0.311 (0.018)	0.355 (0.007)	0.394 (0.002)	0.537 ( < 0.0001)

r, Spearman correlation coefficient; CRP, C-reactive protein; ESR, Erythrocyte sedimentation rate; ESSDAI, EULAR Sjogren’s Syndrome Disease Activity Index; RF, Rheumatoid factor; IgG, Immunoglobulin G; C3, Complement-3; C4, Complement-4.

 Variables with significant correlations with the 14-3-3ƞ protein shown in Table 6 were evaluated with multivariable linear regression analysis regarding their effects on the 14-3-3 ƞ protein. According to the model shown in Table 7, only ESSDAI scores (β = 0.496, 95%CI = 0.079-0.144, *P* = 0.0002) predicted the dependent 14-3-3 ƞ protein variable positively and significantly.

**Table 7:** Multivariable Linear Regression Results between the 14-3-3ƞ Protein and some Study Parameters.

**Parameters**	**Unstandardized Coefficients** **(β-value)**	**Std. Error**	**Standardized Coefficients** **(β-value)**	**95 % CI ** **(Lower-Upper Bound)**		**VIF**	***P*** ** Value**
IgG levels	0.054	0.046	0.137	-0.038	0.146	1.329	0.245
RF leves	0.004	0.005	0.095	-0.007	0.015	1.394	0.429
AntiSSA levels	0.020	0.225	0.013	-0.392	0.431	1.804	0.924
AntiSSB levels	0.392	0.221	0.226	-0.053	0.836	1.606	0.083
ESSDAI scores	0.161	0.041	0.496	0.079	0.244	1.561	0.0002
C3 levels	0.842	1.345	0.072	-1.861	3.546	1.305	0.534
C4 levels	-3.147	2.031	-0.177	-7.277	.934	1.288	0.128
**14-3-3 ƞ protein: **Multiple R = 0.708; R^2^ = 0.502; Adjusted R^2^ = 0.430; Standart error = 1.495, F (7,49) = 7.046, *P* < 0.0001.							

CI, Confidence interval; VIF, Variance ınflation factor; RF, Rheumatoid factor; C3, Complement-3; C4, Complement-4; IgG, Immunoglobulin G; ESSDAI, EULAR Sjogren’s Syndrome Disease Activity İndex.

## Discussion

 The absence of anti-SSA or anti-SSB antibodies in one-third of PSS patients and the inadequacy of markers predicting lymphoma highlight the necessity of investigating new autoantibodies that can be used in diagnosing PSS and predicting lymphoma development. The relationships of the 14-3-3 protein isoforms with neoplastic diseases, RA, and secondary PSS are well known.^25,29-41,43-46^ In this study, we investigated the utility of the 14-3-3η protein as a diagnostic marker, disease activity indicator, and lymphoma predictor in PSS. Our results demonstrated that plasma 14-3-3η protein levels were higher in PSS than healthy controls (*P* < 0.0001), and the cut-off value of 1.741 for the 14-3-3η protein had a sensitivity of 51.9% and a specificity of 84.2% in the diagnosis of PSS (AUC [95% Cl] = 0.758 [0.669–0.848], *P* < 0.0001). In addition, in this study, we found a significant correlation between plasma 14-3-3η protein levels and the disease activity indicator ESSDAI (β = 0.496, 95% CI = 0.079–0.244, *P* = 0.0002). We also showed a substantial correlation between the 14-3-3η protein and hypocomplementemia (β = 0.223, 95% CI = 0.09-1.983, *P* = 0.048), skin purpura (β = 0.252, 95% CI = 0.335–4.903, *P* = 0.022), and high ESSDAI (β = 0.385, 95% CI = 0.318-1.651, *P* = 0.005), which are considered predictors of lymphoma development.

 Although the 14-3-3η proteins in PSS have not been evaluated before, the known mechanisms of action of the 14-3-3 protein isoforms and some pathophysiological processes involved in the pathogenesis of PSS are similar. These similarities make the 14-3-3 proteins a possible candidate to be investigated in the pathogenesis of PSS and lymphoma development. It has been reported that 14-3-3η protein titers are higher in patients with SSS due to SLE, and the 14-3-3η proteins may play a role in the pathogenesis of SSS.^46^ It has been shown that disruption of secretory functions due to apoptosis dysregulation and the development of glandular damage may be a primary mechanism that plays a role in the pathogenesis of PSS.^49^ It has been shown that the 14-3-3η proteins are involved in many cellular functions, including regulation of apoptosis, cell proliferation, and differentiation.^50,51^ Various cytokines such as TNF-α are thought to have essential roles in the pathogenesis of PSS.^52^ The 14 3- 3η proteins have been found to stimulate proinflammatory cytokines such as TNF-α.^53^ Biological abnormalities seen in B lymphocytes play a fundamental role in the pathogenesis of PSS.^54-57^ It has been shown that the 14-3-3 protein isoforms play a critical role in B cell survival and potentially stimulate B cell antibody production.^53^ They also play a role in the pathogenesis of B-cell lymphoma and chemotherapy resistance.^33,58^ The 14-3-3 protein isoforms are associated with decreased survival and poor prognosis in NK/T-cell lymphoma by contributing to asparaginase and gemcitabine resistance through anti-apoptotic mechanisms.^34,35^ In addition, the 14-3-3 protein isoforms contribute to the development of myeloproliferative disease by integrating prosurvival signals in FGFR1 fusion-transformed hematopoietic cells.^59^ In our study, 14-3-3η protein levels were higher in the plasma of PSS patients compared to healthy controls. A significant correlation was found between 14-3-3η protein levels and high ESSDAI and most markers predicting PSS lymphoma.

 Low C4 level, parotid gland swelling, and cryoglobulins as very strong markers; lymphadenopathy, skin purpura/vasculitis, low C3 level, splenomegaly, and high disease activity (ESSDAI) as strong markers; leukopenia, lymphopenia, male gender, RF positivity, anti-SSA/SSB positivity and hypergammaglobulinemia (IgG) as low markers; neutropenia, disease duration, presence of germinal center-like structures in the biopsy, and focus score as uncertain markers have been suggested for the development of lymphoma due to PSS.^60^ Results from the most recent studies investigating the role of predictors in the development of PSS-associated lymphoma suggest a synergistic risk model because they reported that the incidence of lymphoma would increase as the predictive factors present in patients with PSS increase.^10,14,17,61^ In our study, a significant correlation was found between the 14-3-3η protein levels and elevated ESSDAI (*P* = 0.005), hypocomplementemia (*P* = 0.048), and skin purpura (*P* = 0.022).

 Although further prospective studies are needed, the 14-3-3η proteins seem to respond to the need for new markers for PSS that can be used in the diagnosis, disease activity indicator, and prediction of the development of lymphoma, which is the leading cause of mortality.

 The limitations of this study are that it is a case-control study, the salivary gland biopsy ectopic germinal-like structures and focus scores, serum cryoglobulins, and serum beta-2 microglobulins were not evaluated, and the number of patients in some lymphoma predictor subgroups such as parotid gland swelling and purpura were insufficient.

## Conclusion

 This study demonstrated that the 14-3-3η proteins are elevated in the serum of PSS patients. There is a significant relationship between the 14-3-3η proteins and the disease activity indicator ESSDAI and several markers that predict lymphoma development in PSS.
